# All thresholds of maternal hyperglycaemia from the WHO 2013 criteria for gestational diabetes identify women with a higher genetic risk for type 2 diabetes

**DOI:** 10.12688/wellcomeopenres.16097.3

**Published:** 2021-03-23

**Authors:** Alice E. Hughes, M. Geoffrey Hayes, Aoife M. Egan, Kashyap A. Patel, Denise M. Scholtens, Lynn P. Lowe, William L. Lowe Jr, Fidelma P. Dunne, Andrew T. Hattersley, Rachel M. Freathy

**Affiliations:** 1 Institute of Biomedical and Clinical Science, University of Exeter, Exeter, UK; 2 Royal Devon and Exeter Hospitals NHS Foundation Trust, Exeter, UK; 3 Feinberg School of Medicine, Northwestern University, Chicago, IL, USA; 4 Division of Endocrinology, Diabetes and Metabolism, Mayo Clinic School of Medicine, Rochester, MN, USA; 5 Galway Diabetes Research Centre and Saolta Hospital Group, National University of Ireland, Galway, Galway, Ireland; 6 National Institute for Health Research Exeter Clinical Research Facility, Exeter, UK

**Keywords:** Gestational diabetes, genetic scores, fasting plasma glucose, type 2 diabetes

## Abstract

**Background: **Using genetic scores for fasting plasma glucose (FPG GS) and type 2 diabetes (T2D GS), we investigated whether the fasting, 1-hour and 2-hour glucose thresholds from the WHO 2013 criteria for gestational diabetes (GDM) have different implications for genetic susceptibility to raised fasting glucose and type 2 diabetes in women from the Hyperglycemia and Adverse Pregnancy Outcome (HAPO) and Atlantic Diabetes in Pregnancy (DIP) studies.

**Methods: **Cases were divided into three subgroups: (i) FPG ≥5.1 mmol/L only, n=222; (ii) 1-hour glucose post 75 g oral glucose load ≥10 mmol/L only, n=154 (iii) 2-hour glucose ≥8.5 mmol/L only, n=73; and (iv) both FPG ≥5.1 mmol/L and either of a 1-hour glucose ≥10 mmol/L or 2-hour glucose ≥8.5 mmol/L, n=172. We compared the FPG and T2D GS of these groups with controls (n=3,091) in HAPO and DIP separately.

**Results: **In HAPO and DIP, the mean FPG GS in women with a FPG ≥5.1 mmol/L, either on its own or with 1-hour glucose ≥10 mmol/L or 2-hour glucose ≥8.5 mmol/L, was higher than controls (all *P *<0.01). Mean T2D GS in women with a raised FPG alone or with either a raised 1-hour or 2-hour glucose was higher than controls (all *P* <0.05). GDM defined by 1-hour or 2-hour hyperglycaemia only was also associated with a higher T2D GS than controls (all *P* <0.05).

**Conclusions: **The different diagnostic categories that are part of the WHO 2013 criteria for GDM identify women with a genetic predisposition to type 2 diabetes as well as a risk for adverse pregnancy outcomes.

## Introduction

Gestational diabetes mellitus (GDM) has been variably defined since criteria were first developed over 50 years ago ^1^. The World Health Organization (WHO) introduced diagnostic criteria for GDM in 1999, based on criteria for overt diabetes in the general population, with a fasting plasma glucose (FPG) ≥7.0 mmol/L or impaired glucose tolerance with a 2-hour glucose >=7.8 mmol/L post 75 g oral glucose load as part of an oral glucose tolerance test (OGTT), measured between 24 and 28 weeks gestation ^2^. However, lesser degrees of maternal fasting hyperglycaemia have long been associated with a higher risk for adverse perinatal outcomes ^3^, so a FPG ≥6.1 mmol/L (indicative of impaired fasting glycaemia in the non-pregnant population ^4^) was also integrated into the WHO criteria.

The Hyperglycemia and Adverse Pregnancy Outcome (HAPO) Study ^5^ followed 23,316 women who underwent a 2-hour OGTT between 24 and 32 weeks gestation throughout pregnancy and found a continuous association between maternal glucose values and adverse perinatal outcomes, including birth weight ≥90 ^th^ centile (large for gestational age, LGA) and primary caesarean section. In 2010, the International Association of Diabetes and Pregnancy Study Groups (IADPSG) determined cut-off values equivalent to 1.75 times the odds for adverse pregnancy outcomes at mean glucose values, resulting in diagnostic thresholds for FPG ≥5.1 mmol/L, 1-hour glucose ≥10 mmol/L and 2-hour glucose ≥8.5 mmol/L ^6^.

WHO adopted the recommendations of IADPSG in 2013 ^2^, which has resulted in a higher number of cases identified as GDM due to the lower FPG threshold (estimated up to 17.8% prevalence of GDM for IADPSG 2010 criteria ^6^ vs 9.4% prevalence for WHO 1999 criteria ^7^). Whilst these thresholds were chosen for their Obstetric risks, the HAPO Follow-Up Study found that women diagnosed by the newer criteria have a higher risk of developing disorders of glucose metabolism, including T2D, 10 years after the episode of GDM ^8^. A proportion of this risk can be attributed to genetic predisposition, since genome wide association study (GWAS) data from large, non-pregnant population-based studies have identified multiple loci associated with FPG ^9^ and type 2 diabetes ^10^ and some of these are shared with GDM ^11– 16^. Specific to the WHO 2013 criteria, single nucleotide polymorphisms (SNPs) at the *GCK* and *TCF7L2* loci were shown to be associated with FPG and 2-hour glucose levels post-OGTT in women with GDM ^17^. In addition, genetic risk scores for glycaemic traits, including FPG and type 2 diabetes, have been associated with a higher odds for GDM according to the WHO 2013 criteria ^18^. However, it is not known whether the underlying genetic predisposition to fasting hyperglycaemia and type 2 diabetes varies depending on how the diagnosis of GDM is met.

The objective of this study was to investigate whether there is a difference in genetic risk for fasting hyperglycemia and type 2 diabetes according to the different diagnostic thresholds of glucose tolerance from the WHO 2013 criteria for GDM. To do this, we used a genetic score (GS) for FPG (FPG GS) or T2D (T2D GS) (consisting of previously-identified loci ^9, 19^). 

## Methods

### Study population

Women of European ancestry (self-reported white ethnicity) with singleton pregnancies and without known pre-existing diabetes from the Hyperglycemia and Adverse Pregnancy Outcome (HAPO) Study ^5^ ( *n*=2,628) and Atlantic Diabetes in Pregnancy (DIP) study ^20^ ( *n*=1,084) were included. The HAPO study was an observational, multi-centre study ( *N*=23,316 participants from 15 centres) to which women were recruited during pregnancy if they were over 18 years of age ^5^. The 2,665 European-ancestry participants included in the current study were those with genotype data available on selected SNPs (see below). The DIP study had a case-control design: approximately three genotyped control participants without GDM (defined initially as a maternal FPG <5.6 mmol/L and/or 2-hour glucose post oral glucose load <7.8 mmol/L) were available for every genotyped case participant included in our analyses. 

### Sample collection and clinical characteristics

The study methods used in HAPO and DIP have been described in detail previously ^5, 7, 20– 22^. Maternal FPG in mmol/L was measured prior to a standard 2-hour OGTT with 75 g of glucose between 24 and 32 weeks in HAPO and 24 and 28 weeks in DIP. Information on maternal age, pre-pregnancy body mass index (BMI) and systolic blood pressure (SBP, in mmHg) was collected at the OGTT appointment. Clinical characteristics of participants in HAPO and DIP with and without GDM were different (women in DIP were older, had a higher BMI and higher SBP, all *P* <0.01), hence clinical characteristics (where available) have been presented separately.

### GDM diagnostic criteria subgroups

**Figure 1.**
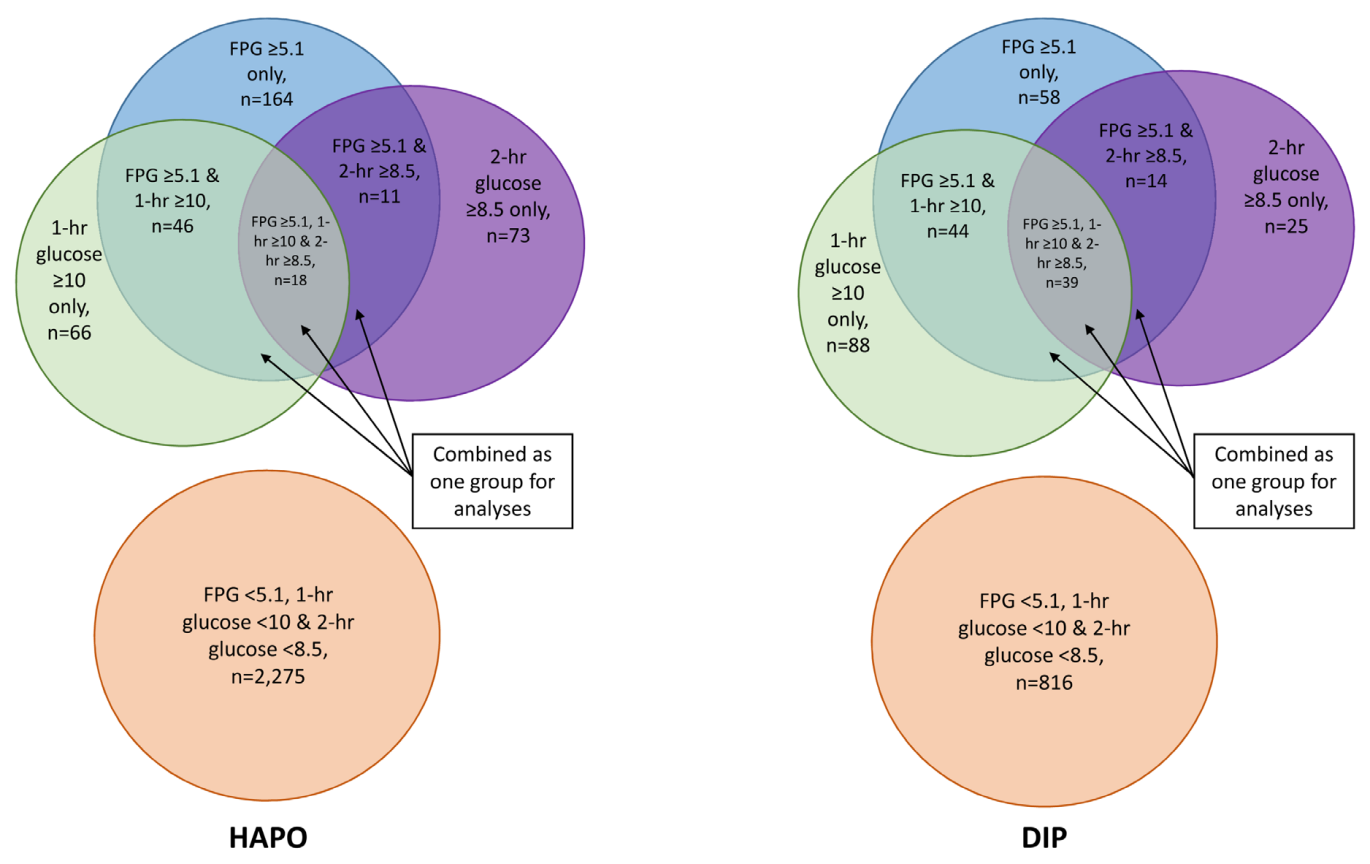
Distribution of participants diagnosed with gestational diabetes (GDM) by different glucose categories in Hyperglycemia and Adverse Pregnancy Outcome Study (HAPO) and Atlantic Diabetes in Pregnancy Study (DIP). All glucose values are in mmol/L. The 1-hour and 2-hour glucose measures refer to the glucose level measured at 1 and 2 hours, respectively, following a 75 g oral glucose load as part of an oral glucose tolerance test. Women with a FPG ≥5.1 mmol/L and either a 1-hour glucose ≥10 mmol/L or 2-hour glucose ≥8.5 mmol/L, or both, were combined as one group for analyses.

We used the WHO 2013 cut-offs (previously IADPSG 2010) to define fasting and 2-hour hyperglycaemia. Thus, in the current study, women diagnosed with GDM were divided into fasting hyperglycaemia only (FPG ≥5.1 mmol/L and 1-hour and 2-hour glucose post 75 g oral glucose load <10 mmol/L and <8.5 mmol/L, respectively, n=222), elevated 1-hour glucose only (1-hour glucose ≥10 mmol/l, FPG <5.1 mmol/L and 2-hour glucose <8.5 mmol/l, n=154), elevated 2-hour glucose only (2-hour glucose ≥8.5 mmol/L, FPG <5.1 mmol/L and 1-hour glucose <10 mmol/L , n=73) and both (FPG ≥5.1 mmol/L and either a 1-hour glucose ≥10 mmol/L or 2-hour glucose ≥8.5 mmol/L, or both, n=172) subgroups Figure 1. Women without GDM were defined as having FPG <5.1 mmol/L, 1-hour glucose <10 mmol/L and 2-hour glucose <7.8 mmol/L (n=3,091). The distributions of the women in the different groups and in each of the study cohorts are shown in Figure 1.

### Genotyping

Genotyping of individual SNPs in DNA samples from both the DIP and HAPO studies was carried out at LGC Genomics (Hoddesdon, UK), using the PCR-based KASP ^TM^ genotyping assay. We first selected 41 SNPs that had been previously associated with type 2 diabetes, and 16 SNPs associated with fasting glucose in non-pregnant individuals, for genotyping in the DIP study. Overlap between the type 2 diabetes and FPG SNPs meant that seven FPG loci were also in the list of type 2 diabetes loci. The median genotyping call rate in the DIP samples was 0.992 (range 0.981–0.996), and there was >99% concordance between duplicate samples (8% of total genotyped samples were duplicates). We excluded one FPG SNP and one type 2 diabetes SNP that showed deviation from Hardy-Weinberg Equilibrium (Bonferroni-corrected *P* value <0.05). For details of included and excluded SNPs and their sources, see Table 1 and Table 2.

**Table 1.** Fifteen SNPs associated with fasting plasma glucose (FPG) and used to construct the FPG genetic score.

Chr:Pos (hg19)	SNP (proxy) ^a^	Locus	Effect/Other Alleles (Proxy)	Effect Allele Frequency (Proxy) ^b^	Beta (mmol/L) ^c^
1:214159256	rs340874	*PROX1*	C/T	0.57	0.013
2:27741237	rs780094	*GCKR*	C/T	0.62	0.029
2:169763148	rs560887	*G6PC2*	C/T	0.70	0.075
3:123065778	rs11708067 (rs2877716)	*ADCY5*	A/G (C/T)	0.75 (0.73)	0.027
3:170717521	rs11920090	*SLC2A2*	T/A	0.88	0.020
7:15064309	rs2191349	*DGKB/* *TMEM195*	T/G	0.55	0.030
7:44235668	rs4607517 (rs1799884)	*GCK*	A/G (T/C)	0.18 (0.18)	0.062
8:118184783	rs13266634	*SLC30A8*	C/T	0.69	0.027
9:4289050	rs7034200	*GLIS3*	A/C	0.48	0.018
10:114758349	rs7903146	*TCF7L2*	T/C	0.29	0.023
11:45873091	rs11605924	*CRY2*	A/C	0.48	0.015
11:47336320	rs7944584	*MADD*	A/T	0.72	0.021
11:61571478	rs174550	*FADS1*	T/C	0.65	0.017
11:92708710	rs10830963	*MTNR1B*	G/C	0.28	0.067
15:62433962	rs11071657	*C2CD4B*	A/G	0.62	0.008

SNP, single nucleotide polymorphism. ^a^Proxy SNPs were genotyped and analysed in the Hyperglycemia and Adverse Pregnancy Outcome Study (both r ^2^ > 0.85 in 340,000 British white unrelated samples from Version 3 release of UK Biobank ^24^, calculated using PLINK software ^25^). ^b^Effect allele frequency was calculated in 340,000 British white unrelated samples from the UK Biobank ^24^. ^c^Beta values were aligned to the trait-raising allele on the + strand (Human Genome Assembly Reference hg19). Source of SNPs and beta values: Dupuis *et al*., 2010 ^9^. We used the same Beta values for proxy SNPs. We excluded rs10885122 ( *ADRA2A* locus) due to deviation from Hardy-Weinberg Equilibrium in the Atlantic Diabetes In Pregnancy Study (Bonferroni corrected *P* < 0.05).

**Table 2.** Thirty-eight SNPs associated with type 2 diabetes (T2D) risk and used to construct the T2D genetic score.

Chr:Pos (hg19)	SNP (proxy) ^a^	Locus	Effect/Other Alleles (Proxy)	Effect Allele Frequency (Proxy) ^b^	Beta ^c^	Source in which SNP was originally identified
1:120526982	rs1493694	*NOTCH2*	T/C	0.11	0.110	Zeggini *et al.*, 2008 ^27^
1:214163675	rs340835	*PROX1*	A/G	0.49	0.062	Dupuis *et al.,* 2010 ^9^
2:27741237	rs780094	*GCKR*	C/T	0.62	0.011	Dupuis *et al.,* 2010 ^9^
2:43732823	rs7578597	*THADA*	T/C	0.89	0.141	Zeggini *et al.*, 2008 ^27^
2:60584819	rs243021	*BCL11A*	A/G	0.46	0.090	Voight *et al.,* 2010 ^26^
2:227093745	rs2943641 (rs2943640)	*IRS1*	C/T (C/A)	0.65 (0.65)	0.083	Rung *et al.,* 2009 ^28^
3:12393125	rs1801282	*PPARG*	C/G	0.88	0.138	Altshuler *et al.,* 2000 ^29^
3:23336450	rs7612463	*UBE2E2*	C/A	0.89	0.102	Yamauchi *et al.,* 2010 ^30^
3:64711904	rs4607103 (rs6795735)	*ADAMTS9*	C/T (C/T)	0.76 (0.59)	0.092	Zeggini *et al.*, 2008 ^27^
3:123065778	rs11708067 (rs2877716)	*ADCY5*	A/G (C/T)	0.75 (0.73)	0.097	Dupuis *et al.,* 2010 ^9^
4:6292915	rs10010131	*WFS1*	G/A	0.60	0.104	Sandhu *et al.,* 2007 ^31^
5:76424949	rs4457053	*ZBED3*	G/A	0.32	0.150	Voight *et al.,* 2010 ^26^
6:20661250	rs7754840 (rs9368222)	*CDKAL1*	C/G (A/C)	0.31 (0.26)	0.170	Zeggini *et al.,* 2007 ^32^
7:28189411	rs1635852	*JAZF1*	T/C	0.49	0.120	Zeggini *et al.*, 2008 ^27^
7:44235668	rs4607517 (rs1799884)	*GCK*	A/G (T/C)	0.18 (0.18)	0.029	Dupuis *et al.,* 2010 ^9^
7:130466854	rs972283 (rs4731702)	*KLF14*	G/A (C/T)	0.51 (0.51)	0.099	Kong *et al.,* 2009 ^33^
8:95937502	rs7845219	*TP53INP1*	T/C	0.50	0.093	Voight *et al.,* 2010 ^4^
8:118184783	rs13266634	*SLC30A8*	C/T	0.69	0.139	Sladek *et al.,* 2007 ^34^
9:22133284	rs10965250	*CDKN2A/B*	G/A	0.83	0.181	Zeggini *et al.,* 2007 ^32^
9:81952128	rs13292136	*CHCHD9*	C/T	0.93	0.182	Voight *et al.,* 2010 ^26^
10:12328010	rs12779790 (rs11257655)	*CDC123/ CAMK1D*	G/A (T/C)	0.18 (0.21)	0.088	Zeggini *et al.*, 2008 ^27^
10:94465559	rs5015480	*HHEX/IDE*	C/T	0.59	0.166	Zeggini *et al.,* 2007 ^32^
10:114758349	rs7903146	*TCF7L2*	T/C	0.29	0.335	Grant *et al.,* 2006 ^35^
11:1696849	rs2334499	*HCCA2/DUSP8*	T/C	0.42	0.080	Kong *et al.,* 2009 ^33^
11:2691471	rs231362	*KCNQ1*	G/A	0.52	0.104	Kong *et al.,* 2009 ^33^
11:2847069	rs163184	*KCNQ1*	G/T	0.48	0.083	Yasuda *et al.,* 2008 ^36^, Unoki *et al.,* 2008 ^37^
11:17408630	rs5215	*KCNJ11*	C/T	0.36	0.089	Gloyn *et al.,* 2003 ^38^
11:72433098	rs1552224	*CENTD2*	A/C	0.84	0.123	Voight *et al.,* 2010 ^26^
11:92673828	rs1387153 (rs10830963)	*MTNR1B*	T/C (G/C)	0.29 (0.28)	0.115	Prokopenko *et al.,* 2009 ^39^, Dupuis *et al.,* 2010 ^9^
12:66170163	rs2612067	*HMGA2*	G/T	0.10	0.180	Voight *et al.,* 2010 ^26^
12:71613276	rs1353362	*TSPAN8/ LGR5*	C/T	0.28	0.103	Zeggini *et al.*, 2008 ^27^
12:121402932	rs7305618 (rs12427353)	*HNF1A*	C/T (G/C)	0.77 (0.81)	0.112	Voight *et al.,* 2010 ^26^
13:80717156	rs1359790	*SPRY2*	G/A	0.71	0.096	Shu *et al.,* 2010 ^40^
15:62396389	rs7172432	*C2CD4A/B*	A/G	0.57	0.068	Yamauchi *et al.,* 2010 ^30^
15:77747190	rs7178572	*HMG20A*	G/A	0.71	0.068	Kooner *et al.,* 2011 ^41^
15:80432222	rs11634397	*ZFAND6*	G/A	0.66	0.102	Voight *et al.,* 2010 ^26^
17:36098040	rs4430796	*HNF1B*	G/A	0.48	0.130	Gudmundsson *et al.*, 2007 ^42^
X:152908152	rs2301142	*DUSP9*	A/G	0.85	0.086	Voight *et al.,* 2010 ^26^

SNP, single nucleotide polymorphism. ^a^Proxy SNPs were genotyped and analysed in the Hyperglycemia and Adverse Pregnancy Outcome Study (r ^2^ > 0.7 in 340,000 British white unrelated samples from Version 3 release of UK Biobank ^24^, except for at *ADAMTS9* where r ^2^ = 0.45 ^24^, calculated using PLINK software ^25^). ^b^Effect allele frequency was calculated in 340,000 British white unrelated samples from the UK Biobank ^24^. ^c^Beta values were aligned to the T2D-risk allele on the + strand (Human Genome Assembly Reference hg19). Beta value = log odds ratio for T2D from genome-wide association study meta-analysis of up to 8130 cases and 38987 controls, published in Voight *et al*. 2010 ^26^. We used the same Beta value for proxy SNPs. We excluded rs8042680 ( *PRC1* locus, Atlantic Diabetes in Pregnancy Study) and rs1470579 ( *IGF2BP2* locus, Hyperglycemia and Adverse Pregnancy Outcome Study) from the T2D GS due to deviation from Hardy-Weinberg Equilibrium (Bonferroni-corrected *P* <0.05). We additionally excluded rs11642841 ( *FTO* locus) due to its primary effect on BMI ^23^.

In the HAPO study, we selected SNPs from the same 16 FPG and 41 type 2 diabetes loci for genotyping in women of European ancestry with DNA available. The selection and genotyping of SNPs in the HAPO study was performed at different times from that in the DIP study. Owing to the differing availability of published GWAS results at these times, the genotyped SNPs differed between HAPO and DIP at 9 of the associated loci. The HAPO SNPs at the nine loci were generally well correlated with those genotyped in DIP (r ^2 ^>0.7, apart from at the *ADAMTS9* locus where r ^2 ^= 0.45). The median genotyping call rate in the HAPO samples was 0.984 (range 0.955–0.991), and the mean concordance between duplicate samples was >98.5% (at least 1% of samples were duplicated). We excluded one SNP that showed deviation from Hardy-Weinberg Equilibrium in the HAPO study (Bonferroni-corrected *P* value <0.05; see Table 1 and Table 2). After exclusion of SNPs that showed deviation from Hardy-Weinberg equilibrium and one SNP from the type 2 diabetes score whose main effect was on BMI (rs11642841 ( *FTO* locus) ^23^, a total of 15 SNPs at FPG-associated loci and 38 SNPs at type 2 diabetes-associated loci were available in both studies for analysis.

### Generating a genetic score for FPG and type 2 diabetes

**Figure 2.**
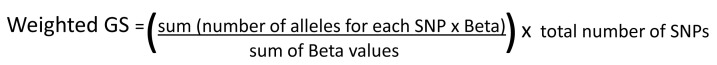
Formula for generating a weighted genetic score (GS). “Number of alleles” corresponds to either the number of risk alleles (type 2 diabetes single-nucleotide polymorphisms (SNPs)) or the number of glucose-raising alleles (fasting plasma glucose SNPs).

Weighted genetic scores for FPG (FPG GS) and type 2 diabetes (T2D GS) were generated using the 15 SNPs and 38 SNPs, respectively. All weights for the FPG GS and T2D GS were taken from Dupuis *et al*. ^9^ and Voight *et al.* ^26^, respectively. The GSs were calculated by taking the sum of the number of FPG-raising or type 2 diabetes risk alleles (0, 1 or 2) for each SNP, multiplied by its corresponding beta value (effect size, see Table 1 and Table 2) for association with FPG or type 2 diabetes, divided by the sum of all beta values and multiplied by the total number of SNPs analysed (see Figure 2 for formula). GS were generated for participants with complete data for all included SNPs only.

### Statistical analyses

***Analysis of clinical characteristics.*** Clinical characteristics were compared between participants with and without GDM in HAPO and DIP using unpaired *t*-tests for normally distributed data and the Wilcoxon Rank-Sum test for non-normally distributed data. *P* values were corrected for 24 comparisons using the Bonferroni method.

***Analysis of associations between FPG GS or T2D GS with glucose levels and GDM.*** Associations of the FPG GS or T2D GS with FPG, 1-hour and 2-hour glucose in women with and without GDM (cases and controls) were analysed using linear regression in HAPO (which was a representative sample of European participants from the whole study cohort) and DIP. *P* values were corrected for 24 comparisons using the Bonferroni method. Our sample of N=2,628 HAPO and N=1,084 DIP participants both provided 100% power to detect associations between fasting glucose levels and the fasting glucose GS at α=0.002, assuming the GS explains 6% variance in glucose levels, which was estimated in 849 pregnant individuals without diabetes in an external study (Exeter Family Study of Childhood Health (EFSOCH)) ^43^. Means for FPG GS and T2D GS in women with and without GDM were compared using unpaired *t*-tests in each study cohort separately, as the genetic scores were higher overall in DIP. *P* values were Bonferroni corrected for 16 comparisons Sensitivity analyses adjusting GS for maternal pre-pregnancy BMI and age (where available) were performed using ANCOVA.

***Statistical software.*** All statistical analyses were performed using Stata version 14.0 (StataCorp LP, College Station, TX, USA). *P-*values <0.05 were considered to indicate evidence of association, unless otherwise stated.

### Ethics approval

Ethics approval was obtained from the Northwestern University Office for the Protection of Research Participants for HAPO (Protocol # 0353-001). The HAPO study protocol was approved by the institutional review board at each field center and all participants gave written, informed consent. Ethics approval was obtained from the local Galway University Hospital Research Ethics Committee for Atlantic DIP (Ref: 54/05) and all participants gave written, informed consent.

## Results

### Clinical characteristics in women with and without GDM

Clinical characteristics for women with and without GDM are summarised in Table 3 for HAPO and DIP, respectively. Women with a FPG ≥5.1 mmol/L (on its own or with either 1-hour or 2-hour hyperglycaemia) had a higher pre-pregnancy BMI than women without GDM in HAPO and DIP ( *P* values <0.001). Women with both fasting and either 1-hour or 2-hour hyperglycaemia were older compared with controls in HAPO ( *P* value <0.05 after Bonferroni correction). In HAPO we observed a higher SBP for women diagnosed with GDM by a FPG ≥5.1 mmol/L only compared with controls ( *P* value <0.001) and they had a higher SBP when either their 1-hour or 2-hour glucose was also raised, but the *P* value was >0.05 after Bonferroni correction. In DIP there was a higher SBP for women diagnosed by both fasting and either 1-hour or 2-hour hyperglycaemia criteria compared with controls ( *P* value <0.05 after Bonferroni correction).

**Table 3.** Clinical characteristics for participants diagnosed with gestational diabetes (GDM) by the different criteria in the Hyperglycemia and Adverse Pregnancy Outcome Study (A) and the Atlantic Diabetes in Pregnancy Study (B).

(A) HAPO					
Variables	Controls with normal glucose	FPG ≥5.1 mmol/L only	1-hr glucose ^a^ ≥10 mmol/L only	2-hr glucose ^a^ ≥8.5 mmol/L only	Both (FPG ≥5.1 mmol/L and either 1-hr glucose ^a^ ≥10 mmol/L or 2-hr glucose ^a^ ≥8.5 mmol/L)
**Median FPG in** **mmol/L (IQR)**	4.5 (4.3-4.7) n=2,275	5.2 (5.1-5.3) n=164	4.8 (4.6-4.9) n=66	4.5 (4.3-4.7) n=48	5.3 (5.2-5.5) n=75
**Median 1-hr glucose** **in mmol/L (IQR)**	7.1 (6.0-8.0) n=2,275	8.4 (7.6-9.2) n=164	10.4 (10.2-11.0) n=66	9.0 (8.6-9.5) n=48	10.6 (10.0-11.2) n=75
**Median 2-hr glucose** **in mmol/L (IQR)**	5.8 (5.1-6.5) n=2,275	6.6 (6.0-7.1) n=164	7.4 (6.6-7.9) n=66	8.9 (8.6-9.1) n=48	7.9 (7.1-8.9) n=75
**Median maternal** **age in years (IQR)**	31 (26-34) n=2,275	31 (27-35) n=164	31 (27-35) n=66	32 (27-34) n=48	32 (29-36) ** n=75
**Median pre-** **pregnancy BMI (IQR)**	22.9 (21.0-26.1) n=2,125	27.5 (23.8-33.1) *** n=142	24.4 (21.2-27.9) n=59	23.0 (20.1-25.1) n=45	28.0 (23.8-35.2) *** n=65
**Median SBP in** **mmHg (IQR)**	108 (102-114) n=2,275	113 (106-119) *** n=164	110 (103-118) n=66	104 (100-116) n=48	110 (103-118) * n=75
**Mean FPG GS (SD)**	15.50 (2.93) n=2,275	16.99 (2.90) *** n=164	16.51 (2.88) * n=66	16.11 (2.06) n=48	16.84 (2.72) ** n=75
**Mean T2D GS (SD)**	41.19 (4.03) n=2,275	42.05 (3.90) * n=164	42.18 (4.15) * n=66	43.40 (4.41) ** n=48	42.74 (5.07) ** n=75
(B) DIP					
Variables	Controls with normal glucose	FPG ≥5.1 mmol/L only	1-hr glucose ^a^ ≥10 mmol/L only	2-hr glucose ^a^ ≥8.5 mmol/L only	Both (FPG ≥5.1 mmol/L and either 1-hr glucose ^a^ or 2-hr glucose ^a^ ≥8.5 mmol/L)
**Median FPG in** **mmol/L (IQR)**	4.3 (4.1-4.5) n=816	5.3 (5.2-5.5) n=58	4.6 (4.4-4.8) n=88	4.5 (4.2-4.7) n=25	5.5 (5.2-5.9) n=97
**Median 1-hr glucose ^a^** **in mmol/L (IQR)**	6.6 (5.6-7.7) n=816	8.7 (7.5-9.1) n=58	10.8 (10.2-11.2) n=88	8.6 (8.1-9.1) n=25	11.2 (10.2-12.0) n=97
**Median 2-hr glucose ^a^** **in mmol/L (IQR)**	5.2 (4.6-6.0) n=816	6.1 (5.5-7.0) n=58	6.9 (5.9-7.8) n=88	8.8 (8.6-9.2) n=25	8.5 (7.5-9.3) n=97
**Median maternal** **age in years (IQR)**	32 (29-36) n=521	35 (31-39) * n=35	34 (31-37) * n=69	32 (29-40) n=16	33 (30-36) n=72
**Median pre-** **pregnancy BMI (IQR)**	25.4 (23.4-28.8) n=454	31.6 (29.0-38.3) *** n=33	29.6 (25.5-35.7) *** n=56	28.5 (25.5-31.1) n=16	33.5 (28.3-37.6) *** n=55
**Median SBP in** **mmHg (IQR)**	117 (108-124) n=437	119 (110-130) n=21	120 (113-130) * n=38	122 (111-134) n=12	120 (115-134) ** n=41
**Mean FPG GS (SD)**	15.76 (2.86) n=816	17.09 (2.86) ** n=58	16.38 (2.94) n=88	16.12 (3.30) n=25	17.60 (3.09) *** n=97
**Mean T2D GS (SD)**	41.73 (3.97) n=816	42.80 (4.16) * n=58	42.75 (4.05) * n=88	44.10 (4.59) ** n=25	43.82 (4.18) *** n=97

BMI, body mass index; DIP, Atlantic Diabetes in Pregnancy Study; FPG, fasting plasma glucose; HAPO, Hyperglycemia and Adverse Pregnancy Outcome Study; IQR, interquartile range; SBP, systolic blood pressure. ^a^The 1-hour and 2-hour glucose measures refer to the glucose level measured at 1 and 2 hours, respectively, following a 75 g oral glucose load as part of an oral glucose tolerance test. ^*^ *P* value <0.05 for comparison with controls (>0.05 after Bonferroni correction). ^**^ *P* value <0.01 for comparison with controls (<0.05 after Bonferroni correction). ^***^ *P* value <0.001 for comparison with controls (remained <0.001 after Bonferroni correction).

### FPG, 1-hour and 2-hour glucose are associated with FPG and T2D GS in pregnant women with and without GDM

FPG, 1-hour and 2-hour glucose values were associated with the fasting and type 2 diabetes genetic scores in HAPO and DIP Table 4. Adjusting for the different measures of glucose tolerance suggested that these associations were not independent of one another.

**Table 4.** Associations for fasting plasma glucose (FPG) and type 2 diabetes (T2D) genetic scores (GS) with different measures of glucose tolerance in women with and without diabetes in the Hyperglycemia and Adverse Pregnancy Outcome Study (A) ^a^ and the Atlantic Diabetes in Pregnancy Study (B) ^b^.

(A) HAPO				
Glucose measure	Beta coefficient (mmol/L) per one unit higher FPG GS (95%CI)	Beta coefficient (mmol/L) per one unit higher FPG GS, with adjustment for other glucose values (95% CI)	Beta coefficient (mmol/L) per one unit higher T2D GS (95% CI)	Beta coefficient (mmol/L) per one unit higher T2D GS, with adjustment for other glucose values (95% CI)
Fasting	0.028 (0.023-0.032) ***	0.022 (0.018-0.027) ***	0.008 (0.004-0.011) ***	0.003 (-3.8 x 10 ^-4^-0.006 )
1-hr ^c^	0.060 (0.040-0.081) ***	0.009 (-0.007-0.025)	0.051 (0.037-0.066) ***	0.019 (0.008-0.031) **
2-hr ^c^	0.032 (0.016-0.048) ***	0.0003 (-0.013-0.013 )	0.034 (0.022-0.045) ***	0.009 (0.00001-0.018)
(B) DIP				
Glucose measure	Beta coefficient (mmol/L) per one unit higher FPG GS (95%CI)	Beta coefficient (mmol/L) per one unit higher FPG GS, with adjustment for other glucose values (95% CI)	Beta coefficient (mmol/L) per one unit higher T2D GS (95% CI)	Beta coefficient (mmol/L) per one unit higher T2D GS, with adjustment for other glucose values (95% CI)
Fasting	0.050 (0.039-0.061) ***	0.030 (0.021-0.039) ***	0.016 (0.008-0.025) **	-0.001 (-0.008-0.006)
1-hr ^c^	0.136 (0.093-0.179) ***	0.034 (-9.35 x 10 ^-6^-0.067 )	0.110 (0.079-0.141 mmol/L) ***	0.055 (0.030-0.078) ***
2-hr ^c^	0.071 (0.038-0.105) ***	-0.015 (-0.042-0.011 )	0.065 (0.041-0.089) ***	0.014 (-0.005-0.033)

CI, confidence interval; DIP, Atlantic Diabetes in Pregnancy Study; HAPO, Hyperglycemia and Adverse Pregnancy Outcome Study. ^a^These analyses were performed in HAPO as it was a representative sample of pregnant women of European ancestry. ^b^As ATLANTIC-DIP had a case-control design the beta coefficients will not be representative of the general pregnant population but are presented for comparison with HAPO. ^c^The 1-hour and 2-hour glucose measures refer to the glucose level measured at 1 and 2 hours, respectively, following a 75 g oral glucose load as part of an oral glucose tolerance test. ^**^ *P* value <0.001, <0.01 after Bonferroni correction. ^***^ *P* value <0.001, remained <0.001 after Bonferroni correction.

### Women diagnosed with GDM by fasting glucose criteria have a higher FPG GS

**Figure 3.**
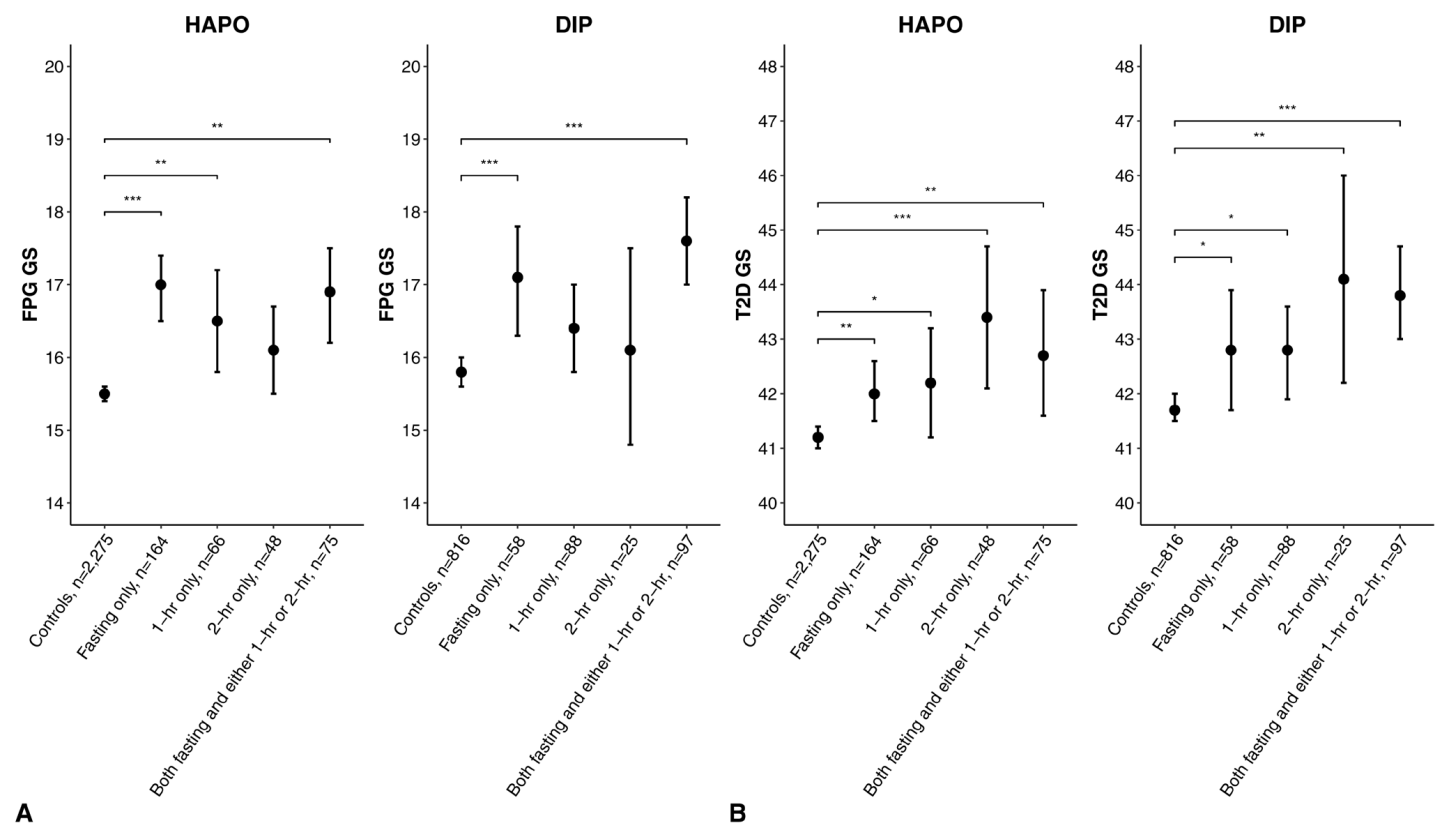
Plots showing mean fasting plasma glucose (FPG) ( **A**) or type 2 diabetes (T2D) ( **B**) genetic score (GS) in each gestational diabetes (GDM) glucose diagnostic category in the Hyperglycemia and Adverse Pregnancy Outcome Study (HAPO) and Atlantic Diabetes in Pregnancy Study (DIP). The 1-hour and 2-hour glucose groups refer to glucose levels measured at 1 and 2 hours, respectively, following a 75 g oral glucose load as part of an oral glucose tolerance test. The control group include women with a FPG <5.1 mmol/L, 1-hour glucose <10 mmol/L and 2-hour glucose <8.5 mmol/L. The fasting only group includes women with a FPG ≥5.1 mmol/L, a 1-hour glucose <10 mmol/L and 2-hour glucose <8.5 mmol/L. The 1-hour only group includes women with 1-hour glucose ≥10 mmol/L, FPG <5.1 mmol/L and 2-hour glucose <8.5 mmol/L. The 2-hour only group includes women with a 2-hour glucose ≥8.5 mmol/L, FPG <5.1 mmol/L and 1-hour glucose <10 mmol/L. The remaining group includes women with both a FPG ≥5.1 mmol/L and either a 1-hour glucose ≥10 mmol/L or 2-hour glucose ≥8.5 mmol/L, or both. Error bars show 95% confidence intervals. * *P* value for comparison between cases and controls <0.05 ** *P* value for comparison between cases and controls <0.01. *** *P* value for comparison between cases and controls <0.001. All *P* values survived Bonferroni correction at α=0.05 except for the FPG GS in women with 1-hour hyperglycaemia in HAPO and the T2D GS in women with isolated fasting or 1-hour hyperglycaemia in HAPO and DIP.

We observed a higher FPG GS in women diagnosed with GDM by fasting hyperglycaemia only and by both fasting and either 1-hour or 2-hour criteria, compared with controls ( Figure 3A, all *P* values for comparison with control group <0.05 after Bonferroni correction). There was also evidence that women with a raised 1-hour glucose only had a higher FPG GS in HAPO ( *P* value for comparison with controls <0.01 but >0.05 with Bonferroni correction), but this was not as strong in DIP ( *P* value =0.05). In contrast, women diagnosed with GDM by 2-hour only criteria did not have a higher FPG GS overall ( *P* values for comparison with controls >0.05 in both studies). Sensitivity analyses adjusted for maternal BMI and age did not materially alter the GS relationships (Extended data Tables 1A and 1B).

### Women diagnosed with GDM by fasting, 1-hour or 2-hour criteria have a higher T2D GS than controls

The T2D GS was higher than controls in women with fasting, 1-hour or 2-hour hyperglycaemia in HAPO and DIP ( Figure 3B): all *P* values for comparison with controls were <0.05 after correction except for the fasting and 1-hour only groups. As with the FPG GS, sensitivity analyses adjusted for maternal BMI and age did not materially affect the associations seen (Extended data Tables 1A and 1B).

## Discussion and conclusions

In this study of 3,712 pregnant women of European ancestry, we have confirmed that women diagnosed with GDM according to the WHO 2013 criteria have a raised genetic risk for type 2 diabetes and shown for the first time that this risk was raised across all of the different measures of glucose tolerance. A genetic predisposition to a higher FPG was present for women who met the fasting glucose criteria (and 1-hour glucose criteria in HAPO), but was not present for women who met the 2-hour criteria.

We confirmed that FPG in pregnant women both with and without GDM was positively associated with a FPG GS generated using SNPs identified in a non-pregnant population ^9^. The 1-hour and 2-hour glucose values were also correlated with the FPG GS, but this could potentially be explained by their association with FPG, since this association was not as strong once this was taken into account. Thus, the observation that the FPG GS was not higher in women diagnosed with GDM due to a 2-hour glucose ≥8.5 mmol/L alone was likely because these women did not have fasting hyperglycemia. However, larger sample sizes would be needed to confidently rule out differences in FPG GS between these groups. Maternal FPG was also associated with the T2D GS, which would be expected, as there are loci within the T2D GS which also raise fasting glucose (e.g. *GCK, MTNR1B*) ^9^. The *ADCY5* locus has also been found to be associated with 2-hour glucose values ^44^. Thus, the observation of a higher T2D GS in women meeting the fasting or 2-hour WHO 2013 criteria for GDM is not surprising. A GWAS for 1-hour glucose values was not available at the time of writing, but since we found the T2D GS to be associated with 1-hour glucose values in HAPO, it is likely that this explains the higher T2D GS seen in the women meeting this criterion for diagnosis of GDM, and will contribute to the higher T2D GS seen in women with both a fasting and either 1-hour or 2-hour hyperglycaemia. However, it is important to note that the relationships between the T2D GS and the different glucose categories did not appear to be independent of one another, and again, although women meeting the diagnosis for GDM in one category may not meet the thresholds for GDM in other categories, they are likely to have a degree of fasting and postprandial hyperglycaemia which will contribute to their higher genetic risk for type 2 diabetes compared with women without GDM.

One might expect that women with both fasting and postprandial hyperglycaemia would have the highest genetic risk for type 2 diabetes, but we did not observe this for the T2D GS in women with both a FPG ≥5.1 mmol/L and either a 1-hour glucose ≥10 mmol/L or 2-hour glucose ≥8.5 mmol/L. On the whole, the relationship between GDM and a higher T2D GS was clearest for women with a raised 2-hour glucose or a combination of raised fasting and 1-hour or 2-hour glucose, but studies with greater statistical power will be needed to confirm whether genetic risk of T2D is heterogeneous across the different thresholds of glucose tolerance that are part of the WHO 2013 criteria for GDM.

Previous studies investigating the association between genetic risk scores for glycaemic traits and GDM have provided interesting insights into the biology of GDM. For example, in a study including women from HAPO as well as a Canadian cohort-study (Gen3G), a fasting glucose genetic risk score was strongly associated with FPG in pregnant women, explaining a similar variance in FPG to the non-pregnant population ^18^. Furthermore, genetic risk scores for insulin sensitivity and insulin secretion were associated with these traits in pregnancy, emphasising there to be an important shared genetic component to these both in and outside of pregnancy. Several SNPs at risk loci included in the genetic scores for FPG and type 2 diabetes risk in this study have previously been associated with GDM at genome-wide significance ( *CDKAL1, G6PC2, GCKR, MTNR1B* ^14, 45^) and at lesser-degrees of significance (e.g. *HNF1A, TCF7L2, HHEX/IDE, PPARG* ^15, 17, 18, 45^). These loci have been implicated in diverse physiological processes influencing glucose metabolism, such as beta cell function and insulin secretion, and insulin resistance secondary to lipodystrophy and disrupted liver lipid metabolism ^46, 47^. Along with previous studies showing associations between GDM and genetic risk scores including SNPs at risk loci associated with type 2 diabetes ^15, 18^, our study supports the growing evidence that genetic determinants of glycaemic traits influence both of these phenotypes.

Although genetic predisposition will contribute to the underlying pathophysiology of GDM, it explains only part of GDM risk. So far, models including genetic risk scores for glycaemic traits have shown limited predictive ability ^18, 48^, suggesting they may not be sufficiently accurate to be used on their own in determining who should be screened for GDM. Therefore, it is still important to consider other well established risk factors, such as parity, maternal age, BMI, ethnicity and socioeconomic background in stratifying risk of GDM ^5^.

This work specifically examining the genetic risk of type 2 diabetes in women diagnosed with GDM according to different measures of glucose tolerance supports the results from the recent HAPO Follow-Up Study ^8^, which showed that women diagnosed with GDM post-hoc according to WHO 2013 criteria had a higher risk for type 2 diabetes 10 to 14 years after pregnancy. We observed the highest BMIs in women diagnosed with GDM by fasting hyperglycaemia only or both criteria, which is consistent with previous research showing that women diagnosed with GDM by the WHO 2013 criteria were more overweight than those diagnosed by WHO 1996 criteria ^7, 49^. However, the associations seen for GDM with FPG GS and T2D GS are not driven by BMI (the genetic variants included within the scores do not primarily affect FPG and T2D risk because of an effect on BMI), suggesting that women with fasting hyperglycaemia in pregnancy are likely to have both BMI-related metabolic factors and a genetic predisposition contributing to type 2 diabetes risk. Furthermore, women with an isolated 2-hour hyperglycaemia did not have a significantly higher BMI than controls, which could suggest a more important role for genetic predisposition in this group of women. However, non-genetic environmental factors related to development of type 2 diabetes such as diet, exercise and socioeconomic deprivation remain a key consideration, as genetics will explain only a portion of risk for type 2 diabetes in women with a history of GDM. For example, a recent study of 2,434 women of white ethnicity found that ~26% women with a history of GDM and a type 2 diabetes genetic risk score in the highest quartile had developed diabetes at follow-up compared with ~23% of women in the lowest genetic risk score quartile ^16^. The GSs in this study were not analysed for their association with incident T2D in the HAPO Follow-Up Study, but this would be useful to establish and make direct comparisons with the results of this work in the future 

In the longer-term, although using the lower FPG threshold from the WHO 2013 criteria for identifying GDM will result in more cases diagnosed, these women will be an important target for long-term follow-up. It is not known whether lifestyle interventions such as guided diet and exercise programmes could modify risk of progressing to type 2 diabetes in women with a history of GDM and a high genetic risk. A study of 1,744 white women suggested that risk of developing type 2 diabetes after a pregnancy affected by GDM was greatest in women with a high genetic risk score and poor diet ^16^. On the other hand, while the Diabetes Prevention Program (DPP) ^50^ trial found that lifestyle intervention or metformin treatment reduced risk of progression to type 2 diabetes in women with impaired glucose tolerance and a history of GDM (according to relevant criteria at time of diagnosis), a genetic risk score for type 2 diabetes did not influence treatment response ^51^. Neither of these studies included women specifically diagnosed by WHO 2013 criteria, but it is clear from this work and that of the HAPO Follow-Up Study that these women would benefit from monitoring after pregnancy and should be considered for targeted lifestyle interventions in public health policies focussing on prevention of type 2 diabetes in adults.

There are limitations of this study that are important to consider. The small number of cases of GDM included has been mentioned and this could have meant that the study was underpowered to show clear differences in T2D GS between the different diagnostic categories. We also studied women from two different studies, where there were notable differences in clinical characteristics, even for women without GDM. Additionally, the FPG and T2D GS were consistently higher in DIP than in HAPO. This is likely to reflect differences in SNPs used to generate the genetic scores and possibly a slightly higher genetic disposition to a raised FPG and type 2 diabetes in DIP. Meta-analysis would have improved power, but was not appropriate due to these differing aspects of each study. However, there were remarkably similar patterns for the genetic score associations amongst the different diagnostic groups in both studies. The results of these analyses are therefore likely to be applicable to women of European ancestry, but further larger-scale studies, including analysis of women with diverse ancestry, will be needed to confirm the associations identified in this study.

In conclusion, women diagnosed with GDM according to the newest WHO 2013 criteria, regardless of how the diagnosis is met, have a higher genetic risk for type 2 diabetes compared with women without GDM. Overall, the criteria identify an important group of women at risk for adverse pregnancy outcomes as well as a higher risk for developing future type 2 diabetes ^8^, which can partly be explained by genetic predisposition. In addition, this study has added to the literature confirming genetic predisposition to type 2 diabetes in women with GDM and supports the call for considering GDM as a key area of investigation in the field of genetics-led precision medicine.

## Data availability

### Underlying data

Data is not freely available due to it consisting of potentially identifiable information, and as such is held securely to protect the interests of research participants in line with the guidance from the relevant ethics committees. However, the ethics committees will allow data analysed and generated in this study to be available to researchers through open collaboration. For access to the data used in this study please contact Dr Rachel Freathy ( r.freathy@exeter.ac.uk) and Professor William Lowe Jr ( wlowe@northwestern.edu) in relation to HAPO and Dr Rachel Freathy and Professor Fidelma Dunne ( fidelma.dunne@nuigalway.ie) in relation to Atlantic DIP. Requests will be reviewed as soon as possible on receipt and will be facilitated with an agreement to ensure that data is transferred and held securely and results of new analyses shared with the relevant study investigators. The websites describing the studies and other data available are https://www.ncbi.nlm.nih.gov/projects/gap/cgi-bin/study.cgi?study_id=phs000096.v4.p1 for HAPO and http://atlanticdipireland.com/for Atlantic DIP.

### Extended data

Figshare: Extended data Wellcome Open Research 16097.pdf. https://doi.org/10.6084/m9.figshare.14180033

The file contains an extended data table with sensitivity analyses adjusting the genetic scores for maternal pre-pregnancy BMI and age and a figure with a directed acyclic graph (DAG) showing how the relationships between the genetic scores and GDM diagnostic category are not driven by maternal pre-pregnancy BMI or age.

## Prior publication

Parts of this work were presented at the European Association for the Study of Diabetes Annual Meeting, Stockholm, Sweden, 14–18 September 2015, the Royal College of Obstetricians and Gynaecologists Annual Academic Meeting, 8–9 February 2018, the East of England Deanery Registrar Prize Meeting, 15 June 2018 and the European Association for the Study of Diabetes Diabetic Pregnancy Study Group Annual Meeting, Graz, Austria, 5–8 September 2019. A previous version of the manuscript was posted as a preprint on bioRxiv, 22 June 2020 (doi: https://doi.org/10.1101/671057).
