# Supplementary material for: All thresholds of maternal hyperglycaemia from the WHO 2013 criteria for gestational diabetes identify women with a higher genetic risk for type 2 diabetes
Source: Wellcome Open Res. 2021 Mar 23;5:175. Originally published 2020 Jul 22. [Version 3] doi: 10.12688/wellcomeopenres.16097.3 (PMC8030121; doi:10.12688/wellcomeopenres.16097.3)
Supplement: Peer review history

# Peer review: Reviewer response for version 3

**DOI:** 10.21956/wellcomeopenres.18435.r43239

**Published:** 2021-04-19

**Authors:** Nael Shaat

**Affiliations:** 1 Department of Endocrinology, Skåne University Hospital, Malmö, Sweden; 2 Department of Clinical Sciences, Genomics, Diabetes and Endocrinology, Lund University, Malmö, Sweden

The authors have addressed my comments properly. I do not have more comments.

Is the work clearly and accurately presented and does it cite the current literature?

Yes

If applicable, is the statistical analysis and its interpretation appropriate?

I cannot comment. A qualified statistician is required.

Are all the source data underlying the results available to ensure full reproducibility?

Yes

Is the study design appropriate and is the work technically sound?

Yes

Are the conclusions drawn adequately supported by the results?

Partly

Are sufficient details of methods and analysis provided to allow replication by others?

Yes

Reviewer Expertise:

Gestational diabetes

I confirm that I have read this submission and believe that I have an appropriate level of expertise to confirm that it is of an acceptable scientific standard.

# Peer review: Reviewer response for version 3

**DOI:** 10.21956/wellcomeopenres.18435.r43237

**Published:** 2021-04-06

**Authors:** Vivian K. Kawai

**Affiliations:** 1 Department of Medicine, Division of Clinical Pharmacology, Vanderbilt University School of Medicine, Nashville, TN, USA

I have no further comments as the authors have provided satisfactory responses to my previous comments.

Is the work clearly and accurately presented and does it cite the current literature?

Partly

If applicable, is the statistical analysis and its interpretation appropriate?

I cannot comment. A qualified statistician is required.

Are all the source data underlying the results available to ensure full reproducibility?

No

Is the study design appropriate and is the work technically sound?

Yes

Are the conclusions drawn adequately supported by the results?

Partly

Are sufficient details of methods and analysis provided to allow replication by others?

Yes

Reviewer Expertise:

risk assessment

I confirm that I have read this submission and believe that I have an appropriate level of expertise to confirm that it is of an acceptable scientific standard.

# Peer review: Reviewer response for version 2

**DOI:** 10.21956/wellcomeopenres.18020.r41368

**Published:** 2021-02-08

**Authors:** Amel Lamri

**Affiliations:** 1 Department of Medicine, McMaster University, Hamilton, ON, Canada

Hughes et al. derived a fasting plasma glucose (FPG) as well as a type 2 diabetes (T2D) genetic risk scores (GS) in participants from the Hyperglycemia and Adverse Pregnancy Outcome (HAPO) and Atlantic Diabetes in Pregnancy (DIP) studies. Pregnant women were assigned to gestational diabetes (GDM) cases or control groups based on their 75g oral glucose tolerance test results using WHO's 2013 criteria. Four different subgroups of cases were devised depending on whether participants exceeded the diagnosis threshold for fasting, 1h-, 2h-glucose measures, or a combination of these. The authors show that FPG GS was lower in GDM controls compared to GDM cases. This association was the strongest when comparing women with a FPG ≥5.1 mmol/L only (1-hour glucose <10 mmol/L and 2-hour glucose <8.5 mmol/L) vs. controls. T2D GS was also lower in GDM controls compared to cases.

 I concur with all the comments from my fellow Reviewers (1 and 2) on this 2 ^nd^ version of the manuscript.

 In addition, I have the following comments/suggestions:

- Tables 1 and 2: Please specify the effect/other allele, effect allele frequency, and beta of the proxy SNPs.
- Table 3: describe the mean and SD of the two GSs in controls as well as in each case group.
- All non-significant results in relation to GS and GDM associations (results shown in Figure 3): There seems to be a big issue with the small samples size in each case group and hence, large SEs. Non-significant results (or results that do not pass multiple testing) are likely due to a lack of statistical power. Similarly, I do not agree with statement (P9 - discussion):” *the observation that the FPG GS was not higher in women diagnosed with GDM due to a 2-hour glucose ≥8.5 mmol/L alone was expected*”. The authors could (and should) meta-analyze results from HAPO and DIP to increase their power. Given the differences between the two studies, adjustments for age and BMI should be considered to reduce between study heterogeneity.

Other Minor Modifications:

- There is no mention in the methods about how LD was estimated in UK Biobank (data version, samples selection, software)
- Table 4: please remove all the mmol/L within the table to facilitate reading, (units can be mentioned in the table’s headers or in the legend).
- Page 9: under “Women diagnosed with GDM by fasting, 1-hour or 2-hour criteria have a higher T2D GS than controls”, please refer to Figure 3B instead of Figure 2B.
- The fact that all weights of T2D GS SNPs were derived from Voigt et al 2010, and that all weights for all FPG GS SNPs were derived from Dupuis et al. 2010 should be mentioned in the methods section under “Generating a genetic score for FPG and type 2 diabetes” (in addition to legends of table 1 and 2) to avoid any confusion with the last column of Table 2.

Is the work clearly and accurately presented and does it cite the current literature?

Yes

If applicable, is the statistical analysis and its interpretation appropriate?

Yes

Are all the source data underlying the results available to ensure full reproducibility?

Partly

Is the study design appropriate and is the work technically sound?

Yes

Are the conclusions drawn adequately supported by the results?

Partly

Are sufficient details of methods and analysis provided to allow replication by others?

Partly

Reviewer Expertise:

Genetics and genomics of complex cardio-metabolic traits

I confirm that I have read this submission and believe that I have an appropriate level of expertise to confirm that it is of an acceptable scientific standard, however I have significant reservations, as outlined above.

# Response

**Published:** 2021-03-08

**Authors:** Alice Hughes

**Affiliations:** University of Exeter, UK

We would like to thank reviewer 3 for their detailed comments on version 2 of the manuscript. We have addressed their points in turn below:

 1. **Tables 1 and 2: Please specify the effect/other allele, effect allele frequency, and beta of the proxy SNPs.**

 We have provided the effect/other alleles, EAF and beta for the proxy SNPs in the Tables 1 and 2 as requested. We used the same beta value for proxy SNPs and have clarified this in the table footnote. Thank you for alerting us to this.

 2. **Table 3: describe the mean and SD of the two GSs in controls as well as in each case group.**

 We have provided the mean and SD of the GSs to Table 3 as requested.

 3. **All non-significant results in relation to GS and GDM associations (results shown in Figure 3): There seems to be a big issue with the small samples size in each case group and hence, large SEs. Non-significant results (or results that do not pass multiple testing) are likely due to a lack of statistical power. Similarly, I do not agree with statement (P9 - discussion):” the observation that the FPG GS was not higher in women diagnosed with GDM due to a 2-hour glucose ≥8.5 mmol/L alone was expected”. The authors could (and should) meta-analyze results from HAPO and DIP to increase their power. Given the differences between the two studies, adjustments for age and BMI should be considered to reduce between study heterogeneity.**

 We agree with the reviewer’s comment that the wide confidence intervals and non-significant P values after adjustment for multiple testing is likely due to the small sample sizes and resulting low power. We mentioned the small sample size could lead to less distinct associations between the diagnostic categories in the eighth paragraph of the discussion, but have clarified this to include the fact that this could be related to low power. It now reads: “The small number of cases of GDM included has been mentioned and this could have meant that the study was underpowered to show clear differences in T2D GS between the different diagnostic categories”.

 Regarding the comment on it being expected that there was not a higher FPG GS in individuals with a raised 2-hour glucose, we agree that in the context of a small study, that this might represent the sample size, rather than a considerably lower genetic predisposition to raised fasting glucose. However, we do think it remains the case that women who meet the criteria for GDM because of a raised 2-hour glucose alone but not a raised fasting glucose will likely have a lower FPG GS than women who meet the criteria because of fasting hyperglycaemia, and that was supported by the results in both cohorts. We have changed the wording of this to reflect these points to: “Thus, the observation that the FPG GS was not higher in women diagnosed with GDM due to a 2-hour glucose ≥8.5 mmol/L alone was likely because these women did not have fasting hyperglycemia. However, larger sample sizes would be needed to confidently rule out differences in FPG GS between these groups.”

 Meta-analysis of the results from both cohorts would improve power, and we did intend to do this when we set-out to complete this work. However, due to the different structures of the separate studies (in HAPO a random subset were genotyped, whilst DIP genotyped three women without GDM for every one woman diagnosed with GDM using the clinical criteria at the time of recruitment), different SNPs (proxies were used in the HAPO GS, please see the Methods section for more details), higher GSs in the DIP cohort and different proportions of women in the different diagnostic categories, we felt that meta-analysis would not be combining like-for-like. We do think that the similar patterns of association support the findings in both studies, but for the reasons mentioned we took the decision not to meta-analyse. These reasons are shown in paragraph 8 of the discussion, but we agree that a comment making it clear that these were reasons precluding meta-analysis should be added and have done so in paragraph 8 of the discussion: “Meta-analysis would have improved power, but was not appropriate due to these differing aspects of each study”.

 4. **There is no mention in the methods about how LD was estimated in UK Biobank (data version, samples selection, software)**

 Thank you for pointing this important detail about LD estimates out. We have added to the footnotes of Tables 1 and 2 how LD was calculated.

 5. **Table 4: please remove all the mmol/L within the table to facilitate reading, (units can be mentioned in the table’s headers or in the legend).**

 We have edited Table 4 to remove the multiple instances of mmol/L to help make it more readable.

 6. **Page 9: under “Women diagnosed with GDM by fasting, 1-hour or 2-hour criteria have a higher T2D GS than controls”, please refer to Figure 3B instead of Figure 2B.**

 Thank you for pointing out this typo, we have corrected it accordingly.

 7. **The fact that all weights of T2D GS SNPs were derived from Voigt et al 2010, and that all weights for all FPG GS SNPs were derived from Dupuis et al. 2010 should be mentioned in the methods section under “Generating a genetic score for FPG and type 2 diabetes” (in addition to legends of table 1 and 2) to avoid any confusion with the last column of Table 2.**

 Thank you for pointing this out. We have added a sentence to that effect in the Methods section (please see response 2 to reviewer 1 for more details).

# Peer review: Reviewer response for version 2

**DOI:** 10.21956/wellcomeopenres.18020.r39654

**Published:** 2020-12-07

**Authors:** Nael Shaat

**Affiliations:** 1 Department of Endocrinology, Skåne University Hospital, Malmö, Sweden; 2 Department of Clinical Sciences, Genomics, Diabetes and Endocrinology, Lund University, Malmö, Sweden

Authors have investigated whether the fasting, 1-hour and 2-hour glucose thresholds from the WHO 2013 criteria for GDM have different genetic susceptibility to raised fasting glucose and type 2 diabetes in women from 2 cohorts, the Hyperglycemia and Adverse Pregnancy Outcome (HAPO) and Atlantic Diabetes in Pregnancy (DIP) studies. For this purpose, they used genetic scores for fasting plasma glucose (FPG GS) and type 2 diabetes (T2D GS). The main findings were that the mean FPG GS and mean T2D GS were higher in women with only a FPG ≥5.1 mmol/L (or combined with 1- or 2-h glucose) than in controls. In addition, mean T2D GS in women with a raised FPG alone or with either a raised 1-hour or 2-hour glucose was higher than controls. Authors concluded that women with a history of GDM have a higher risk of type 2 diabetes in later-life and the risk differs according to how the diagnosis of GDM is met. 

 The paper is interesting. Many questions have already been raised by the other reviewer.

 I have 2 comments:

1. The authors should mention the statistical power of the study.
2. I agree with the other reviewer that the analyses should be adjusted for BMI even if the authors have excluded SNPs associated with BMI.

Is the work clearly and accurately presented and does it cite the current literature?

Yes

If applicable, is the statistical analysis and its interpretation appropriate?

I cannot comment. A qualified statistician is required.

Are all the source data underlying the results available to ensure full reproducibility?

Yes

Is the study design appropriate and is the work technically sound?

Yes

Are the conclusions drawn adequately supported by the results?

Partly

Are sufficient details of methods and analysis provided to allow replication by others?

Yes

Reviewer Expertise:

Gestational diabetes.

I confirm that I have read this submission and believe that I have an appropriate level of expertise to confirm that it is of an acceptable scientific standard, however I have significant reservations, as outlined above.

# Response

**Published:** 2021-03-08

**Authors:** Alice Hughes

**Affiliations:** University of Exeter, UK

We would like to thank reviewer 2 for their time in helping to improve the manuscript. We have addressed their two points as follows:

 1. **The authors should mention the statistical power of the study. **The expected size of any genetic score difference between the case and control groups was unknown. However, as an indicative power calculation, our sample of N=2,628 HAPO and N=1,084 DIP participants both provided 100% power to detect associations between fasting glucose levels and the fasting glucose GS at alpha=0.002, assuming the GS explains 6% variance in glucose levels, which was estimated in 849 pregnant individuals without diabetes in an external study (Exeter Family Study of Childhood Health (EFSOCH)). We have added this information to the Methods under the subheading “Analysis of associations between FPG GS or T2D GS with glucose levels and GDM.” of the “Statistical analyses” section.

 2. **I agree with the other reviewer that the analyses should be adjusted for BMI even if the authors have excluded SNPs associated with BMI.**

 We have addressed adjustments for BMI in response to point number 3 of reviewer 1.

# Peer review: Reviewer response for version 2

**DOI:** 10.21956/wellcomeopenres.18020.r41234

**Published:** 2020-11-12

**Authors:** Vivian K. Kawai

**Affiliations:** 1 Department of Medicine, Division of Clinical Pharmacology, Vanderbilt University School of Medicine, Nashville, TN, USA

I have the following comments for the reviewed manuscript:

1. SNPs for FPG risk score in Table 1 comes from Dupuis et al., 2010 study, which is OK since this is probably the largest GWAS for fasting glucose in individuals of European ancestry.
2. However, table 2 shows that selected T2D variants came from different studies and populations. Effect estimates depend largely on the sample size and the allele frequency of the variant in a population, as well as the impact of a variant in specific population ancestries. Thus, the authors need to comment how this approach could have affect their results and why they select this approach over using a non-weighted GRS or using only estimates from the largest study in European ancestry regardless of the p-value reported. An alternative approach is to use one of the cohort to define the estimates for each variant and then use this estimates in the second cohort to calculate the GRS.
3. The authors argue that BMI is unlikely to explain the association observed between GDM and the risk scores because the variants included into the GRS has no effect on BMI. They also say that adjusting for BMI has the potential to bias estimates since patients with higher risk scores are likely to developed GDM at lower BMI, which is true. However, confounding is a major problem in genetic studies and with the data shown, we cannot rule out that BMI is a major driven of the results, which undermines the study.  A sensitivity analysis including pre-pregnancy BMI and maternal age is recommended as well as a discussion of how including BMI in the analysis may affect the estimates.
4. Although this was not the scope of the study, the authors should add a short comment why the performance of these GRS for T2D in the HAPO and DIP cohort was not studied, particularly when they mentioned that women in HAPO cohort with GDM had higher risk for T2D 10 to14 years after pregnancy. This is a question that probably every reader would have.

Is the work clearly and accurately presented and does it cite the current literature?

Partly

If applicable, is the statistical analysis and its interpretation appropriate?

I cannot comment. A qualified statistician is required.

Are all the source data underlying the results available to ensure full reproducibility?

No

Is the study design appropriate and is the work technically sound?

Yes

Are the conclusions drawn adequately supported by the results?

Partly

Are sufficient details of methods and analysis provided to allow replication by others?

Yes

Reviewer Expertise:

risk assessment

I confirm that I have read this submission and believe that I have an appropriate level of expertise to confirm that it is of an acceptable scientific standard, however I have significant reservations, as outlined above.

# Response

**Published:** 2021-03-08

**Authors:** Alice Hughes

**Affiliations:** University of Exeter, UK

We would like to thank reviewer 1 for reviewing the second version of the manuscript, having already commented on the first version. We have made changes to improve the manuscript based on the new comments and have addressed them below:

 1. **SNPs for FPG risk score in Table 1 comes from Dupuis et al., 2010 study, which is OK since this is probably the largest GWAS for fasting glucose in individuals of European ancestry.**Yes, the SNPs and effect size estimates part of the FPG genetic score come from one study (Dupuis et al.)

 2. **However, table 2 shows that selected T2D variants came from different studies and populations. Effect estimates depend largely on the sample size and the allele frequency of the variant in a population, as well as the impact of a variant in specific population ancestries. Thus, the authors need to comment how this approach could have affect their results and why they select this approach over using a non-weighted GRS or using only estimates from the largest study in European ancestry regardless of the p-value reported. An alternative approach is to use one of the cohort to define the estimates for each variant and then use this estimates in the second cohort to calculate the GRS.**

 We did not use effect size estimates from different studies. The effect sizes were all derived from one GWAS (Voight et al., which was the biggest and most recently available study at the time of genotyping). This is shown by the superscript “c” in the “Beta” column. The studies in the last column refer to where the SNP was originally identified. Reviewer 3 picked up on this being unclear and we have taken their advice to include a sentence under “Generating a genetic score for FPG and type 2 diabetes” in the Methods section that reads: “All weights for the FPG GS and T2D GS were taken from Dupuis et al. ^9^ and Voight et al. ^26^, respectively.

 3. **The authors argue that BMI is unlikely to explain the association observed between GDM and the risk scores because the variants included into the GRS has no effect on BMI. They also say that adjusting for BMI has the potential to bias estimates since patients with higher risk scores are likely to developed GDM at lower BMI, which is true. However, confounding is a major problem in genetic studies and with the data shown, we cannot rule out that BMI is a major driven of the results, which undermines the study.  A sensitivity analysis including pre-pregnancy BMI and maternal age is recommended as well as a discussion of how including BMI in the analysis may affect the estimates.**

 BMI may confound genetic estimates due to collider bias, as we stated in our previous response. We would not expect the relationships between GS and GDM diagnosis to change according to maternal BMI and age as the SNPs are not causally associated with higher BMI or age. The directed acyclic graph (DAG, found in Extended data file: https://doi.org/10.6084/m9.figshare.14180033) illustrates this.

 However, since all reviewers have requested it, we have performed analyses adjusted for maternal pre-pregnancy BMI and age (where available) and present the adjusted GS means and 95% confidence intervals for each GDM diagnostic criteria and controls in an Extended Data table which can be found at the following link: https://doi.org/10.6084/m9.figshare.14180033

 The estimates and associations are very similar to those shown in the main manuscript (see Figure 3; to aid comparison we have added a column in the Extended data table with the unadjusted values). Since BMI and maternal age were not available in all participants (93% of HAPO participants and 51% of DIP participants), the sample size for the adjusted analyses was smaller and there was a corresponding attenuation of the strength of associations (as shown by wider confidence intervals). However, as can be seen by comparing the adjusted and non-adjusted GSs, the estimates were within the 95% CI of the non-adjusted associations, indicating that they were consistent between the adjusted and non-adjusted analyses. We would prefer not to include this data in the main manuscript for the reasons discussed above and in our previous response, but we have included the sentences “sensitivity analyses adjusted for maternal BMI and age did not materially alter the GS relationships (Extended data Tables 1A and 1B)” and “As with the FPG GS, sensitivity analyses adjusted for maternal BMI and age did not materially affect the associations seen (Extended data Tables 1A and 1B).” in the “Women diagnosed with GDM by fasting glucose criteria have a higher FPG GS” and “Women diagnosed with GDM by fasting, 1-hour and 2-hour criteria have a higher T2D GS than controls” sections of the results, respectively.

 4. **Although this was not the scope of the study, the authors should add a short comment why the performance of these GRS for T2D in the HAPO and DIP cohort was not studied, particularly when they mentioned that women in HAPO cohort with GDM had higher risk for T2D 10 to14 years after pregnancy. This is a question that probably every reader would have.**

 The individuals genotyped in this study do not completely crossover with individuals included in the HAPO Follow-Up Study and since the follow-up time has been short so far, we are unlikely to have the power to study the performance of the T2D score with incident T2D in this cohort, although this is something that would be valuable to do in the future. We agree that a comment on this would be helpful and have added the following sentence to the discussion at the end of the sixth paragraph “The GSs in this study were not analysed for their association with incident T2D in the HAPO Follow-Up Study, but this would be useful to establish and make direct comparisons with the results of this work in the future”.

# Peer review: Reviewer response for version 1

**DOI:** 10.21956/wellcomeopenres.17665.r40254

**Published:** 2020-09-25

**Authors:** Vivian K. Kawai

**Affiliations:** 1 Department of Medicine, Division of Clinical Pharmacology, Vanderbilt University School of Medicine, Nashville, TN, USA

The manuscript by Hughes A.E. and collaborators titled: All thresholds of maternal hyperglycaemia for the WHO 2013 criteria for gestational diabetes identify women with a higher genetic risk for type 2 diabetes” test the hypothesis that each diagnosed criteria have different genetic risk using a FPGGS and a T2D GS.

 I have the following comments:

1. Please clarify what is the objective of the study and the clinical utility.
2. Previous studies have shown the limited utility GRS for T2 (and likely FPG) in the detection of women at risk for GDM, suggesting that either other genetic factors are the main driver of GDM or environmental exposures. The authors did not provide a convincing explanation in their discussion of the relevance of their findings.
3. Having an increase genetic risk for T2D or fasting hyperglycemia is irrelevant if the authors cannot show what are the clinical implications of this genetic susceptibility. It is known that women with GDM are at higher risk for developing T2D in the future, but the real question is whether a high genetic susceptibility or other clinical factors are the main drivers of this risk in women who already experienced GDM.
4. A major drawback of using GS is that they cannot provide mechanistic insights, so it is unclear how the use of GS for T2D and FPG for GDM can provide any relevant information in GDM.
5. Please provide a more comprehensive explanation for the GS calculation.
6. What is the definition of European ancestry: self-reported white or was based on population stratification analysis.
7. Pre-pregnancy BMI and maternal age are well known risk factors for GDM. I suggest supporting the statement that the association seen between the GS and the different diagnostic criteria are not driven by BMI with data. We cannot assume that variants have the same impact in BMI variability in non-pregnant and pregnant populations.
8. Table 4, please confirm that the outcomes follow normal distribution and provide results for DIP

Is the work clearly and accurately presented and does it cite the current literature?

Partly

If applicable, is the statistical analysis and its interpretation appropriate?

I cannot comment. A qualified statistician is required.

Are all the source data underlying the results available to ensure full reproducibility?

No

Is the study design appropriate and is the work technically sound?

Yes

Are the conclusions drawn adequately supported by the results?

Partly

Are sufficient details of methods and analysis provided to allow replication by others?

Yes

Reviewer Expertise:

risk assessment

I confirm that I have read this submission and believe that I have an appropriate level of expertise to state that I do not consider it to be of an acceptable scientific standard, for reasons outlined above.

# Response

**Published:** 2020-10-13

**Authors:** Alice Hughes

**Affiliations:** University of Exeter, UK

We thank the reviewer for their constructive comments on this work. With the help of these comments we have taken the opportunity to improve the manuscript. We have addressed their comments in turn below and updated the manuscript accordingly (Version 2).

**1. Please clarify what is the objective of the study and the clinical utility.** 

 Thank you for prompting us to clarify the message of the paper by stating the objective of the study. The objective of the study was to investigate whether there was a difference in genetic risk for fasting hyperglycaemia and type 2 diabetes according to the different diagnostic thresholds of glucose tolerance as part of the WHO 2013 criteria for gestational diabetes (GDM). We originally stated this in the form of a hypothesis to test in the last paragraph of the introduction, but we have updated the introduction to refocus it as an objective of the study instead (paragraph 4 of the “Introduction” section).

 In relation to the clinical utility of the study there are several points to consider. The thresholds for fasting, 1-hour and 2-hour glucose in the WHO 2013 criteria were chosen based on their Obstetric risks (i.e. primary caesarean section, large for gestational age baby, HAPO study reference: https://doi.org/10.1056/NEJMoa0707943). Although it is well known that women with a history of GDM have a higher risk of type 2 diabetes in later-life, it is not known if this differs according to how the diagnosis of GDM is met, and whether this may partly reflect underlying predisposing genetic factors. As expected, we observed a higher genetic risk for fasting hyperglycaemia in women who had a met the fasting plasma glucose criteria ( >5.1 mmol/L). We observed a higher genetic risk for type 2 diabetes across all diagnostic categories. Therefore, these criteria identify an important group of women with a genetic risk for type 2 diabetes. This is important for the clinical community to be aware of, since there are many different criteria for GDM used across the world which utilise different criteria (for example, in the United Kingdom the fasting glucose cut-off is 5.6 mmol/L and in Denmark a fasting glucose is not included). Our findings support the findings of the HAPO Follow-Up Study, which showed women diagnosed with GDM according to the WHO 2013 criteria had higher rates of disorders of glucose metabolism 10 years later (Reference: https://doi.org/10.1001/jama.2018.11628). The genetic risk is of particular interest for women who meet the criteria for GDM due to a high 2-hour glucose, as we did not observe a significantly higher BMI in this group of women. Therefore, if they had not been identified as having GDM, these women may not necessarily be considered a high-risk group who would require monitoring and follow-up (e.g. with an annual HbA1c). We have added to the discussion to emphasise the potential implications for long-term follow-up (paragraph 6, “Discussion and conclusion” section).

 Of course, it is not known whether preventative lifestyle interventions could modify this genetic risk; we touch on this in the discussion in relation women with a history of GDM in the Diabetes Prevention Program (DPP) trial (Reference: https://dx.doi.org/10.2337%2Fdc13-0700). A recent study suggested that a genetic score for type 2 diabetes was more strongly associated with developing type 2 diabetes in women with a history of GDM and a poor diet (Reference: https://dx.doi.org/10.1136%2Fbmjdrc-2019-000850). But, they also point out that the relationship between the T2D genetic risk score and incident type 2 diabetes was modest. We have added to this area of the discussion to provide a more balanced discussion on the potential implications of a high genetic risk for type 2 diabetes, emphasising the uncertainty of whether the genetic risk could be of benefit in targeting public health interventions in relation to post-GDM care (paragraphs 6 and 7).

**2. Previous studies have shown the limited utility GRS for T2 (and likely FPG) in the detection of women at risk for GDM, suggesting that either other genetic factors are the main driver of GDM or environmental exposures. The authors did not provide a convincing explanation in their discussion of the relevance of their findings.**

 We agree that the genetic risk for fasting hyperglycaemia and type 2 diabetes will only explain a portion of risk for GDM. The genetic risk will not explain risks associated with BMI, maternal age, parity and socioeconomic deprivation. In addition, these genetic scores will not explain the higher risks for GDM seen in women of African and South Asian heritage, for example. Whilst this study did not aim to assess the power of the genetic scores to diagnose or predict GDM, we have added to the discussion taking into account these considerations and referencing studies that have focussed on this as a new paragraph in the discussion (paragraph 5 of “Discussion and conclusion” section). 

**3. Having an increase genetic risk for T2D or fasting hyperglycemia is irrelevant if the authors cannot show what are the clinical implications of this genetic susceptibility. It is known that women with GDM are at higher risk for developing T2D in the future, but the real question is whether a high genetic susceptibility or other clinical factors are the main drivers of this risk in women who already experienced GDM**.

 We have partially responded to the clinical implications in response to comment 1 and point to the updated version of the discussion. Similar to comment number 2, we agree that genetic risk is likely to explain only a portion of risk for type 2 diabetes and we have added to the discussion to emphasise the importance of other factors (please see paragraph 6 of the “Discussion and conclusion” section and the response to comment 1 above). However, we also believe that genetic risk for type 2 diabetes and fasting hyperglycaemia are likely to explain part of the relationship between GDM and type 2 diabetes in later-life. We discuss this in more detail in paragraphs 4 and 6 of the “Discussion and conclusion” section.

**4. A major drawback of using GS is that they cannot provide mechanistic insights, so it is unclear how the use of GS for T2D and FPG for GDM can provide any relevant information in GDM.**

 We believe that genetic scores provide important insights into the biology of GDM. For example, the risk loci included in the scores have been implicated in beta cell function, proinsulin secretion and impaired insulin action secondary to unfavourable metabolic patterns of adiposity and liver lipid metabolism (References: https://doi.org/10.1371/journal.pmed.1002654 and https://doi.org/10.1038/s41588-018-0084-1). The relevance of this to GDM was underlined in a recent paper by Powe et al. (Reference: https://doi.org/10.2337/db18-0203), which showed associations between genetic risk scores for fasting glucose, fasting insulin, and insulin secretion and sensitivity with GDM. In particular, they also observed strong associations between a fasting glucose GS and fasting glucose in pregnancy, similar to the associations seen outside of pregnancy, emphasising an important shared genetic predisposition. We did not seek to repeat the biological relevance of these associations demonstrated in this paper in our work, but rather show that there is a higher genetic risk for type 2 diabetes associated with the different measures of glucose tolerance in the WHO 2013 criteria for GDM. However, we agree that a discussion considering the likely underlying biology of genetic scores in relation to type 2 diabetes and GDM will help add to the context of the paper and have included a new paragraph in relation to this in the discussion (paragraph 4).

**5. Please provide a more comprehensive explanation for the GS calculation.**

 We have provided a description of the genetic score (GS) calculation in the Methods section under the heading titled “Generating a genetic score for FPG and type 2 diabetes”. We have also included the formula used in Figure 2. We have added a sentence referring to Tables 1 and 2 which show where the beta (effect size) was obtained for each SNP used in the score to clarify this further.

**6. What is the definition of European ancestry: self-reported white or was based on population stratification analysis.**

 European ancestry was based on self-reported white ethnicity in both HAPO and DIP. We have added this to the Methods section under the heading titled “Study population”.

**7. Pre-pregnancy BMI and maternal age are well known risk factors for GDM. I suggest supporting the statement that the association seen between the GS and the different diagnostic criteria are not driven by BMI with data. We cannot assume that variants have the same impact in BMI variability in non-pregnant and pregnant populations.**

 The SNPs at the risk loci included in the genetic scores influence glycaemic traits independent of BMI. We did not include SNPs which had their main effect on BMI (e.g. *FTO*). As BMI is not on the causal pathway between the genetic score and the outcome (GDM diagnostic criterion) it is not necessary to adjust for it in analyses, hence why it is not included in the data presented in this paper. Adjusting for BMI also has the potential to bias estimates due to collider-stratification bias (Reference: https://doi.org/ 10.1093/hmg/ddw433). For example, we observe a paradoxical negative correlation between the T2D GS and pre-pregnancy BMI in HAPO (Spearman’s Rho -0.05, *P* value 0.01). This is not a true association and comes about because individuals with a higher genetic risk score are more likely to develop type 2 diabetes at a lower BMI than individuals with a lower genetic risk score. However, we agree that there are key differences in GDM risk according to BMI, which we have shown in the comparison of BMI between the different diagnostic criteria in Table 3 summarising the clinical characteristics of the participants in the studies. We have expanded on the importance of the role of BMI contributing to risk of type 2 diabetes and GDM in the discussion (see responses to Comments 1 and 3 and paragraphs 5 ad 6 of the “Discussion and conclusions” section).

**8. Table 4, please confirm that the outcomes follow normal distribution and provide results for DIP.**

 Fasting glucose, 1-hour and 2-hour glucose have a positive skew in both HAPO and DIP (hence their presentation of median with IQR in Table 3). A normal distribution of outcomes is not a pre-requisite for linear regression models (Reference: https://doi.org/10.1017/CBO9780511790942), so we do not think this is of concern for this analysis, but we are happy to supply additional information if the reviewer would like this.

 We have now included a second part to the table with the associations in DIP (which had a similar pattern of associations to HAPO), but with a cautionary note (footnote b to Table 4) that the beta coefficients seen in a case-control study design will not be applicable to a general pregnant population due to the relative over-representation of cases of GDM. Now we have included these analyses *P* values for association have been corrected for 24 comparisons and we have updated the Methods section under the heading “Statistical analyses”.
